# Sintilimab treatment for chronic active Epstein–Barr virus infection and Epstein–Barr virus-associated hemophagocytic lymphohistiocytosis in children

**DOI:** 10.1186/s13023-023-02861-9

**Published:** 2023-09-22

**Authors:** Ruyue Chen, Qiang Lin, Yun Zhu, Yunyan Shen, Qinying Xu, Hanyun Tang, Ningxun Cui, Lu Jiang, Xiaomei Dai, Weiqing Chen, Xiaozhong Li

**Affiliations:** Department of Nephrology and Immunology, Children’s Hospital of Soochow University, No.303 Jing De Road, Gusu District, Suzhou, 215002 Jiangsu China

**Keywords:** Epstein–Barr virus (EBV), Chronic active Epstein–Barr virus infection (CAEBV), Epstein–Barr virus-associated hemophagocytic lymphohistiocytosis (EBV-HLH), Programmed cell death 1 (PD-1), Sintilimab

## Abstract

**Background:**

Chronic active Epstein–Barr virus infection (CAEBV) and Epstein–Barr virus-associated hemophagocytic lymphohistiocytosis (EBV-HLH) are rare but life-threatening progressive diseases triggered by EBV infection. Glucocorticoid/immunosuppressants treatment is temporarily effective; however, most patients relapse and/or progress. Hematopoietic stem cell transplantation (HSCT) is a potentially curative therapy; however, there are risks of transplantation-associated complications. Currently there is no standard treatment for CAEBV and EBV-HLH. Programmed death protein 1 (PD-1) inhibitors have achieved a high response in many EBV-related diseases. Sintilimab (a recombinant human IgG4 monoclonal antibody against PD-1) disrupts the interaction between PD-1 and its ligand, leading to T cell reinvigoration.

**Methods:**

A retrospective analysis was performed on three children with CAEBV or EBV-HLH in the Children’s Hospital of Soochow University between 12 December 2020 and 28 November 2022. The efficacy of sintilimab was evaluated.

**Results:**

Three patients, including two males and one female, were analyzed. Among them, two children were diagnosed with CAEBV with intermittent fever for more than four years, and one child was diagnosed with EBV-HLH. After sintilimab treatment and a mean follow-up of 17.1 months (range 10.0–23.3 months), patients 1 and 3 achieved a complete clinical response and patient 2 achieved a partial clinical response. All three children showed a > 50% decrease in EBV-DNA load in both blood and plasma. EBV-DNA copies in sorted T, B, and NK cells were also markedly decreased after sintilimab treatment.

**Conclusion:**

Our data supported the efficacy of PD-1 targeted therapy in certain patients with CAEBV and EBV-HLH, and suggested that sintilimab could provide a cure for these diseases, without HSCT. More prospective studies and longer follow-up are needed to confirm these conclusions.

## Background

The infection rate of Epstein–Barr virus (EBV) in the population worldwide is more than 95%, and the impaired balance between the host immune response and EBV can lead to various EBV-associated lymphoproliferative disorders (LPDs) of B, T, or natural killer (NK) cells [1, 2]. Chronic active EBV infection (CAEBV) and EBV-associated hemophagocytic lymphohistiocytosis (EBV-HLH) are rare but life-threatening diseases. To date, a standard treatment approach for CAEBV and EBV-HLH has not been established. Conventional therapies, including antiviral drugs and immune-modulatory agents, can lead to temporary remission; unfortunately, most patients relapse and progress [3]. Etoposide-based HLH-1994 and HLH-2004 regimens are widely used; however, some patients are refractory or intolerant to intensive chemotherapy [4, 5]. Hematopoietic stem cell transplantation (HSCT) was considered as the only potentially curative method; however, it led to numerous transplantation-associated complications [3].

Programmed cell death-1 (PD-1) is a representative immunosuppressive checkpoint and is mainly expressed in activated T cells, B cells, NK cells, macrophages, dendritic cells, monocytes, and myeloid cells, and in immune-privileged sites [6]. The interaction between PD-1 and its ligands leads to inhibition of T cell proliferation, activation, cytokine production, and cytotoxic T lymphocyte killer functions [7–9]. In chronic infections or tumors, lasting antigen-exposure leads to permanent PD-1 expression, which can limit immune-mediated clearance of pathogens or neoplastic cells [10]. The overexpression of PD-1 on virus-specific T cells has been documented in EBV and other virus infections [11, 12]. Immune evasion via the PD-1 pathway has been confirmed to play an important role in various EBV-positive cancers [13, 14]. PD-1 inhibition has achieved a remarkable response in EBV-positive lymphoma and EBV-associated gastric cancer, in which it is believed to reverse EBV or cancer-mediated immunosuppression by restoring immunity and releasing T cells [15–19]. However, there have been few reports of the treatment of CAEBV and EBV-HLH with PD-1 inhibitors [20–23]. Sintilimab is a recombinant human IgG4 monoclonal antibody against PD-1 that disrupts the interaction between PD-1 and its ligand, leading to T cell reinvigoration [16]. The present study discussed the use of sintilimab in CAEBV and EBV-HLH combined with clinical experience in three patients.

## Methods

We retrospectively analyzed the clinical data of three children diagnosed with CAEBV or EBV-HLH who were treated with sintilimab in the Department of Nephrology and Immunology, Children’s Hospital of Soochow University between 12 December 2020 and 28 November 2022. Sintilimab was provided by the Xinda Biopharmaceutical (Suzhou, China) company. Real-time fluorescent quantitative PCR and TaqMan hydrolysis probes were used to detect EBV-DNA in peripheral blood and plasma. Intracellular EBV-DNA copies in sorted peripheral blood mononuclear cells (PBMCs) were also determined using quantitative PCR.

We used previously described criteria for response assessment [20, 24, 25]. A clinical complete response (clinical CR) was defined as the resolution of all clinical signs and symptoms, including fever, liver dysfunction, progressive skin lesions, or vasculitis, accompanied by a significant decrease in EBV-DNA. Resolution of some of the above symptoms was defined as a clinical partial response (clinical PR). A molecular complete response (molecular CR) comprised a significant decrease in EBV-DNA load in both blood and plasma (< 10^2.5^ copies/mL). A 50% drop in EBV-DNA load in either blood or plasma was defined as a molecular partial response (molecular PR).

## Results

### Case 1

A Chinese male aged 6 years and 2 months (bodyweight, 16 kg) was admitted to our department with intermittent fever accompanying elevated liver enzymes for more than 4 years. The child had visited other hospitals many times and relevant examinations showed increased EBV-DNA copies in his peripheral blood and plasma, accompanying hepatosplenomegaly, liver function abnormalities, and lymphadenopathy (Table 1). There were no significant improvements in his symptoms and signs after treatment with antiviral drugs, intravenous immunoglobulin (IVIG), and glucocorticoids. Upon admission to our hospital, high EBV DNA loads in his peripheral blood, plasma, and bone marrow (1.97 × 10^6^ copies/mL, 3.15 × 10^4^ copies/mL, and 2.25 × 10^5^ copies/mL, respectively) were observed. Intracellular EBV-DNA copies in sorted PBMCs were also high (CD3+CD4+ T cells: 7.7 × 10^5^ copies/mL, CD3+CD8+ T cells: 1.1 × 10^5^ copies/mL, CD3-CD19+ B cells: 1.2 × 10^4^ copies/mL, and CD56+ NK cells: 2.7 × 10^6^ copies/mL, respectively). Whole exome sequencing (WES) revealed no clear genetic mutations. The virus capsid antigen (VCA) IgG antibody, EBV nuclear antigen (EBNA) IgG antibody, and early antigen IgG antibody tests were positive. The VCA IgM antibody test was negative (Table 2). The above clinical manifestations and examination results met the recently revised diagnostic criteria for CAEBV [26].

**Table 1:** Baseline characteristics before sintilimab treatment

Case	Sex	Age (m)	Weight (kg)	Clinical manifestation	Disease duration	Primary treatment	Diagnosis
1	M	74	16	Intermittent fever Hepatosplenomegaly liver function abnormalities lymphadenopathy	More than 4 years	Antiviral drugs IVIG Methylprednisolone	CAEBV
2	F	150	26.7	Intermittent fever Intermittent skin rash Oral ulcer lymphadenopathy	More than 6 years	Antiviral drugs	CAEBV
3	M	50	15.5	Intermittent fever Intermittent skin rash Mild abdominal pain lymphadenopathy	More than 1 month	Antiviral drugs IVIG Methylprednisolone pulse	EBV-HLH

*IVIG* Intravenous immunoglobulin, *CAEBV *Chronic active Epstein–Barr virus infection, *EBV-HLH* Epstein–Barr virus-related hemophagocytic lymphohistiocytosis

**Table 2:** EBV DNA loads and EBV-specific antibodies before sintilimab treatment

Case	EBV DNA loads (copies/ml)			EBV specific antibody				
	Peripheral blood	Plasma	Bone marrow	Viral capsid antigen IgG antibody	Capsid antigen IgG antibody affinity	Viral capsid antigen IgM antibody	Early antigen IgG antibody	Nuclear antigen IgG antibody
1	1.97 × 10^6^	3.15 × 10^4^	2.25 × 10^5^	+	High	−	+	+
2	1.41 × 10^7^	2.19 × 10^4^	5.34 × 10^3^	+	High	−	+	+
3	1.11 × 10^6^	2.73 × 10^3^	−	+	High	−	−	+

The patient received treatment with sintilimab combined with ganciclovir (or oral valganciclovir), methylprednisolone (3.75 mg/kg/day and then gradually reduced), cyclosporine, IVIG (1 g/kg), and rituximab (140 mg/m^2^). Sintilimab was administered via intravenous infusion at 3 mg/kg every 3–6 weeks (Table 3).

**Table 3:** Sintilimab combined with other treatments

Case	Sintilimab			Glucocorticoids	Immunosuppressants	Antiviral drugs	Other main treatment
	Dose	Frequency	Cycles				
1	3 mg/kg	3–6 weeks	30	Methylprednisolone	Cyclosporine	Ganciclovir (or oral valganciclovir)	IVIG Rituximab
2	3 mg/kg	3 weeks	13	Prednisone acetate	Tacrolimus	Oral valganciclovir	–
3	3 mg/kg	3 weeks	20	Methylprednisolone	Cyclosporine	Ganciclovir (or oral valganciclovir)	IVIG

**Fig. 1:**
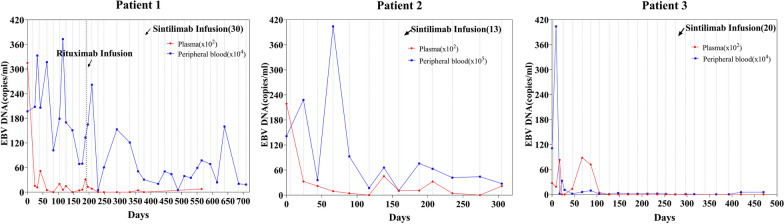
Epstein–Barr virus (EBV)-DNA copies in plasma and peripheral blood

**Fig. 2:**
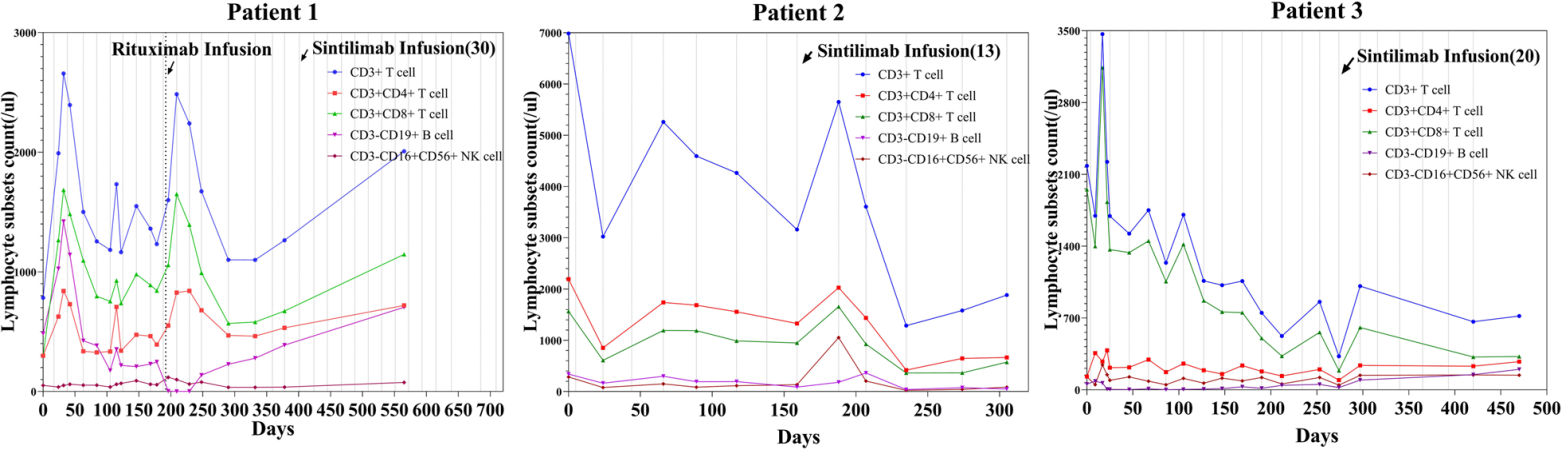
Lymphocyte subset counts before and after sintilimab treatment

During a follow-up of 23.3 months, fever and relevant signs improved significantly. When the 13th and 14th cycle interval was lengthened to 6 weeks, he developed fever again; however, the child's body temperature returned to normal and stabilized when the medication interval returned to 3 weeks. EBV-DNA loads in peripheral blood and plasma decreased markedly after sintilimab treatment (Fig. 1). EBV-DNA loads in sorted PBMCs decreased markedly, especially in CD3+CD8+ T cells (Table 4). The CD3+ T cell counts were maintained at a normal level, and the levels of CD3+CD8+ T cells were higher than those of CD3+CD4+ T cells (Fig. 2). After treatment with 30 cycles of sintilimab, patient 1 achieved clinical CR and molecular PR, and methylprednisolone and cyclosporine were gradually stopped.

**Table 4:** EBV-DNA copies in sorted T, B, and NK cells before and after sintilimab treatment

EBV-DNA (copies/10^6^ cells)	Case 1		Case 2		Case 3	
	Before	After 12 cycles	Before	After 4 cycles	Before	After 7 cycles
CD3+CD4+ T cells	7.7 × 10^5^	2.9 × 10^4^	3.0 × 10^4^	1.0 × 10^4^	–	–
CD3+CD8+ T cells	1.1 × 10^5^	7.0 × 10^2^	3.5 × 10^4^	2.2 × 10^4^	–	–
CD3−CD19+ B cells	1.2 × 10^4^	1.8 × 10^3^	2.3 × 10^4^	1.1 × 10^4^	6.5 × 10^5^	1.6 × 10^5^
CD56+ NK cells	2.7 × 10^6^	5.4 × 10^4^	5.4 × 10^4^	1.4 × 10^4^	1.8 × 10^4^	–

### Case 2

A Chinese girl aged 12 years and 6 months (body weight, 26.7 kg) was admitted to our department with intermittent fever accompanying skin rash and oral ulcer lasting more than 6 years (Table 1). The child had visited other hospitals many times, and her condition had not improved. Upon admission to our hospital, her EBV-DNA copies in peripheral blood, plasma, and bone marrow were elevated (1.41 × 10^7^ copies/mL, 2.19 × 10^4^ copies/mL and 5.34 × 10^3^ copies/mL, respectively), and intracellular EBV-DNA copies in sorted PBMCs were positive (CD3+CD4+ T cells: 3.0 × 10^4^ copies/mL, CD3+CD8+ T cells: 3.5 × 10^4^ copies/mL, CD3−CD19+ B cells: 2.3 × 10^4^ copies/mL, and CD56+ NK cells: 5.4 × 10^4^ copies/mL, respectively). WES revealed no genetic mutations. The VCA IgG antibody and EBNA IgG antibody tests were positive. The VCA IgM antibody test was negative (Table 2). The above clinical manifestations and examination results met the recently revised diagnostic criteria for CAEBV [26].

She accepted the combined therapy of sintilimab, prednisone acetate (1 mg/kg/day and then gradually reduced), valganciclovir and tacrolimus. Sintilimab was administered via intravenous infusion at 3 mg/kg every 3 weeks (Table 3).

She never developed a fever; her skin rash and oral ulcer decreased gradually without recurrence during 10 months of follow-up. We observed 80.6% and 90.1% decreases in EBV-DNA copies in peripheral blood and plasma, respectively (Fig. 1), and a > 37% decrease in intracellular EBV-DNA copies (Table 4). The CD3+ T cell counts increased, and the levels of CD3+CD4+ T cells were higher than those of CD3+CD8+ T cells (Fig. 2). After regular treatment with 13 cycles of sintilimab, patient 2 achieved clinical PR and molecular PR. Prednisone acetate was reduced to a low dose (2.5 mg/day) and tacrolimus was stopped after the 13th cycle.

### Case 3

A Chinese boy aged 4 two years and 2 months (bodyweight, 15.5 kg) was admitted to the intensive care unit for intermittent high fever accompanying skin rash and mild abdominal pain for over one month (Table 1). The clinical manifestation and examination, including high fever, hepatosplenomegaly, anemia (hemoglobin < 90 g/L), thrombocytopenia (platelet < 100 × 10^9^/L), neutropenia (neutrophil < 1.0 × 10^9^/L), hyperferritinemia (> 500 ng/mL), hypertriglyceridemia (> 2.65 g/L), hemophagocyte observed on marrow smear, and high EBV DNA loads in peripheral blood and plasma (1.11 × 10^6^ copies/mL and 2.73 × 10^3^ copies/mL, respectively), suggested a diagnosis of EBV-HLH based on the HLH-2004 diagnostic criteria (Table 2). Intracellular EBV-DNA was mainly present in CD3-CD19+ B cells and CD56+ NK cells (6.5 × 10^5^ copies/mL and 1.8 × 10^4^ copies/mL, respectively). No familial HLH-related gene mutations were detected. No significant improvement in his fever and relevant signs were observed after treatment with antiviral drugs, IVIG, and methylprednisolone pulse.

His parents refused transplantation; therefore, he was transferred to our department for sintilimab treatment combined with the application of ganciclovir (or oral valganciclovir), methylprednisolone (20 mg/kg/day × 2 d, 5 mg/kg/day and then gradually reduced), cyclosporine, and IVIG. Sintilimab was administered via intravenous infusion at 3 mg/kg every 3 weeks (Table 3).

The body temperature of the child improved significantly. He had a transient fever again before the 3rd and 6th cycles. EBV-DNA in plasma fell from 2.73 × 10^3^ copies/mL to an undetectable level and remained stable after six cycles of sintilimab (Fig. 1). EBV-DNA copies in CD56+ NK cells decreased from 1.8 × 10^4^ copies/mL to an undetectable level after the 7th cycle (Table 4). The CD3+ T cell counts were maintained at normal and above levels, and the levels of CD3+ CD8+ T cells were higher than those of CD3+CD4+ T cells (Fig. 2). After regular treatment with 20 cycles of sintilimab and a follow-up of 18.1 months, patient 3 achieved clinical CR and molecular PR. Currently, all drugs had been stopped except for regular IVIG every month.

## Discussion

In this retrospective clinical data analysis, we reviewed the clinical and immunological characteristics of three children with CAEBV or EBV-HLH. After sintilimab treatment and a mean follow-up of 17.1 months (range 10.0–23.3 months), two patients achieved a clinical CR and one achieved a clinical PR. All three children showed a > 50% decrease in EBV-DNA load in both blood and plasma, which suggested a molecular PR. EBV-DNA copies in sorted PBMCs were also significantly decreased.

After primary EBV infection, individuals develop robust EBV-specific T cell immune responses, with EBV-specific CD8+ and some CD4+ T cells functioning as cytotoxic T cells, defending against the virus [27]. Strong T cell immunity plays a key role in controlling infection by decreasing the viral load and eliminating infected cells. However, the continuous viral antigen burden during the course of chronic viral infection leads to persistent stimulation of antigen-specific T cells, resulting in T cell exhaustion [10, 28]. Studies have found that patients with CAEBV have a large number of myeloid-derived suppressor cells (MDSCs) that decrease the function of effector T cells, resulting in persistent uncontrolled EBV infection [29]. EBV-induced HLH is the most common subtype of secondary virus-associated HLH during childhood, and is characterized by uncontrolled activation of T lymphocytes and macrophages [27]. Kasahara et al. reported that EBV infection was predominant in CD8+ T cells in patients with EBV-HLH, whereas the dominant EBV-infected cell populations in patients with CAEBV were non-CD8+ lymphocyte subpopulations [30]. Analysis of PBMCs in some patients with EBV-HLH showed a reduction in CD4+ T cells and abnormal activation of CD8+ T cells [27, 31].

T cell activation relies mainly on a two-signal model [32]. The first signal confers specific recognition of cognate antigenic peptides presented by major histocompatibility complex (MHC) molecules and the T cell receptor (TCR). The second signal comprises co-stimulatory and co-inhibitory signals, which modulate TCR signaling positively or negatively to direct T cell function [32, 33]. A group of inhibitory or stimulatory molecules expressed on immune cells, antigen-presenting cells, tumor cells, or other types of cells are regarded as immune checkpoints [34]. PD-1 (also known as PDCD1 and CD279) is a representative immunosuppressive checkpoint that plays a key role in programmed death signaling to regulate T cell-mediated responses [35]. PD-1 is activated by binding to programmed cell death 1 ligand 1 (PD-L1) or programmed cell death 1 ligand 2 (PD-L2) [36]. Upon ligand binding, SH2 domain-containing protein tyrosine phosphatase 2 (SHP-2) is recruited to the immunoreceptor tyrosine-based switch motif (ITSM) of PD-1, leading to SHP-2 dephosphorylation of different targets downstream of TCR [7, 9].

Tumor cells exert immune escape because of the abnormal immune surveillance mediated by immune checkpoints. Studies have reported overexpression of PD-L1 mRNA and protein in EBV-driven malignant tumors, such as EBV-associated gastric cancer, Hodgkin's lymphoma and EBV-peripheral T-cell lymphoma, mediated by interferon gamma (IFN-γ), mitogen activated protein kinase (MAPK), nuclear factor kappa B (NF-κB), and signal transducer and activator of transcription 3 (STAT3) signaling pathways [13, 14, 37–40]. Inhibitors targeting the PD-1 pathway can rescue T cells from an exhausted state and revive the immune response against EBV and cancer cells [7]. Recent reports showed that PD-1 inhibitors achieved a remarkable response in EBV-positive lymphoma and EBV-associated gastric cancer [15–19].

Similarly, high PD-1 expression on virus-specific T cells has been observed in infections with lymphocyte choriomeningitis virus (LCMV), human immunodeficiency virus (HIV), hepatitis B virus (HBV), and hepatitis C virus (HCV) [11, 41]. An increase in the frequency of PD-1 on the surface of CD8+ T cells was also found during symptomatic primary EBV infection, and was associated with elevated EBV loads [12]. However, reports of CABEV and EBV-HLH treated with PD-1 inhibitors are rare. You et al. [21] reported that sintilimab helped to treat mixed chimeric and reactivated EBV in a patient with adult-onset CAEBV after allo-HSCT, in which EBV-DNA was ultimately undetectable and a stable donor chimerism was obtained. Ma et al. [22] reported that 16 patients with CAEBV were treated with PD-1 inhibitors, including pembrolizumab (2/16), sintilimab (9/16), or nivolumab (5/16), and 12 patients responded to PD-1 inhibitors. Song et al. [23] investigated the combination therapy of sintilimab and lenalidomide in patients with CAEBV and reported an overall response rate of 54.2%. Liu et al. [20] reported that seven patients with relapsed/refractory EBV-HLH were treated with nivolumab, among which 71.4% of the patients reached clinical CR. Clinical studies support the efficacy of PD-1 targeted therapy in a subset of patients with CAEBV and EBV-HLH.

In summary, our findings suggested that the PD-1 inhibitor sintilimab could achieve remarkable results in pediatric patients with CAEBV and EBV-HLH, and might provide a cure for these disease without the use of HSCT. PD-1 inhibition might restore immunity and release T cells, providing benefits for patients with CAEBV and EBV-HLH. However, further clinical trials and mechanistic studies are needed to verify its effectiveness.

## Data Availability

The authors declare that the data supporting the findings of this study are available within the paper. Should any raw data files be needed in another format, they are available from the corresponding author upon reasonable request. Source data are provided with this paper.

## Abbreviations

EBV: Epstein–Barr virus
CAEBV: Chronic active Epstein–Barr virus infection
EBV-HLH: Epstein–Barr virus-associated hemophagocytic lymphohistiocytosis
HSCT: Hematopoietic stem cell transplantation
PD-1: Programmed death protein 1
LPDs: Lymphoproliferative disorders
PBMCs: Peripheral blood mononuclear cells
CR: Complete response
PR: Partial response
IVIG: Intravenous immunoglobulin
VCA: Virus capsid antigen
EBNA: EBV nuclear antigen
MDSCs: Myeloid-derived suppressor cells
MHC: Major histocompatibility complex
TCR: T cell receptor
PD-L1: Programmed cell death 1 ligand 1
PD-L2: Programmed cell death 1 ligand 2
SHP-2: SH2 domain-containing protein tyrosine phosphatase 2
ITSM: Immunoreceptor tyrosine-based switch motif
IFN-γ: Interferon gamma
MAPK: Mitogen activated protein kinase
NF-κB: Nuclear factor kappa B
STAT3: Signal transducer and activator of transcription 3
LCMV: Lymphocyte choriomeningitis virus
HIV: Human immunodeficiency virus
HBV: Hepatitis B virus
HCV: Hepatitis C virus
